# Is Absolute Pitch Associated With Musical Tension Processing?

**DOI:** 10.1177/2041669520971655

**Published:** 2020-11-18

**Authors:** Jun Jiang*, Tang Hai, Dongrui Man, Linshu Zhou

**Affiliations:** Music College, 12544 Shanghai Normal University, Shanghai, China; School of Life Sciences, 34747 Shanghai University, Shanghai, China; Music College, 12544 Shanghai Normal University, Shanghai, China

**Keywords:** absolute pitch, musical tension, music ability, mode, key

## Abstract

Absolute pitch (AP) is a superior ability to identify or produce musical tones without a reference tone. Although a few studies have investigated the relationship between AP and high-level music processing such as tonality and syntactic processing, very little is known about whether AP is related to musical tension processing. To address this issue, 20 AP possessors and 20 matched non-AP possessors listened to major and minor melodies and rated the levels of perceived and felt musical tension using a continuous response digital interface dial. Results indicated that the major melodies were perceived and felt as less tense than the minor ones by AP and non-AP possessors. However, there was weak evidence for no differences between AP and non-AP possessors in the perception and experience of musical tension, suggesting that AP may be independent of the processing of musical tension. The implications of these ﬁndings are discussed.

Absolute or perfect pitch (hereafter AP) is the ability to name or produce isolated musical notes in the absence of a reference note (Deutsch, 2013; Ward, 1999). It is often viewed as a sign of extraordinary music ability considering that many famous musicians (e.g., Mozart, Beethoven, and Heifetz) are AP possessors (Deutsch, 2002; Deutsch et al., 2004; Petran, 1932). However, some researchers suspect that AP might be musically irrelevant (Levitin & Rogers, 2005; Miyazaki, 2004a), because there is no evidence to suggest that AP possessors are more musically gifted than non-AP musicians (Patel, 2008).

Investigations into musical advantages of AP have produced mixed results. Some researchers found that AP possessors outperformed non-AP possessors on interval identification (Dooley & Deutsch, 2011), musical dictation (Dooley & Deutsch, 2010), and melody discrimination in C major key (Miyazaki & Rakowski, 2002). Nevertheless, others suggested that AP possessors did not perform better than non-AP possessors in identifying interval categories (Miyazaki, 1993) and chord types (Ziv & Radin, 2014) and discriminating paired melodies (Miyazaki, 2004b). These inconsistent results may result from whether the tasks employed in those studies required the strategy of relative pitch, an ability to make pitch judgments about the relation between notes (Zatorre et al., 1998). Because the tasks in those studies (Miyazaki, 1993, 2004b; Ziv & Radin, 2014) require relative pitch, the induced Stroop-like interference may have reduced performance of AP possessors (Dooley & Deutsch, 2011).

Noteworthily, all of the aforementioned studies have focused on low-level music processing. Although a few studies have examined the role of AP in higher level music processing such as tonality and syntax processing, the results remain uncertain. Marvin and Brinkman (2000) showed that AP possessors relative to non-AP possessors were good at identifying the keys of musical excerpts. On the contrary, Temperley and Marvin (2008) found no difference in the key identification between AP and non-AP possessors. Likewise, Jiang et al. (2010) found that AP possessors performed better than non-AP possessors on the tasks of melodic and harmonic syntax, while Coll et al. (2019) demonstrated that AP possessors did not have increased sensitivity to harmonic syntax compared with non-AP possessors. These contradictory results may be attributed to the fact that the low-level pitch naming strategy (i.e., AP or relative pitch) can be directly used to recognize the key or ending tone of the music (e.g., Coll et al., 2019; Marvin & Brinkman, 2000). Therefore, to exclude the effect of low-level musical processing, tasks unrelated to pitch naming should be adopted when investigating the association between AP and higher level musical processing.

Musical tension, one of the core aspects of higher level music processing, is another way to understand the music ability of AP possessors. Musical tension is closely related to expectation processes, in particular to the buildup of expectancy, the violation of expectancy, the anticipation for resolution, and the resolution (Koelsch, 2012), and thus taps into emotional responses to music (Huron, 2006; Juslin, 2013; Lehne & Koelsch, 2015; Meyer, 1956). Despite being influenced by psychoacoustical features such as loudness, tempo, timbre, and sensory dissonance (Farbood, 2012; Farbood & Price, 2017; Koelsch, 2014), the general tension-resolution pattern of music is primarily governed by harmonic and melodic structure (Lehne et al., 2013). However, to date, no research has examined whether AP possession is linked with musical tension processing. The goal of the present work, therefore, was to examine the relationship between AP and the processing of musical tension.

To this aim, the present study compared the ratings of musical tension in AP and non-AP possessors. First, to induce greater tension responses (Lerdahl & Krumhansl, 2007; Wong et al., 2009), we manipulated melodic regularities at the end of melodies, where melodies ended on all 11 tones but the leading tone of a major or minor scale. Second, given that a music passage in the minor mode is more dissonant and unpleasant (Parncutt, 2014; Virtala & Tervaniemi, 2017) and thus tenser (Fang et al., 2017; Huynh et al., 2017) than that in the major mode, melodies in major and minor modes were included. Third, given that tonal expectancies are stronger when melody sequences were presented in the same key than in the different keys from trial to trial (Jiang et al., 2016), melodies in this study were presented in single-key and mixed-key conditions to induce different levels of musical tension. Furthermore, we included tension perception and experience tasks to assess the relationship between perceived (Gingras et al., 2016) and felt (Lehne et al., 2013) musical tension. If AP is relevant to tonal music structure processing (Jiang et al., 2010; Marvin & Brinkman, 2000), we hypothesized that AP and non-AP possessors would give different ratings to perceived and felt musical tension. On the basis of the findings described previously (e.g., Fang et al., 2017; Parncutt, 2014), we hypothesized that minor melodies would be rated as tenser than major ones.

## Method

### Participants

Eighty-four instrumental music students with and without self-reported AP were recruited from Shanghai Normal University to take two AP tests as in the study by Baharloo et al. (1998). In AP test 1, 40 pure tones from C2 to G#8 were used; in AP test 2, 40 piano tones from C1 to G#7 were used. Based on the standard pitch of A4 (440 Hz), each tone was created from the 12-tone equal temperament scale with a duration of 1 s. In each test, 40 tones were divided into four blocks, and each block consisted of 10 trials, with a 3-s interval between trials. Tones were presented in pseudorandom order, with the restriction that two successive tones were separated by more than two octaves and a semitone and were different in pitch names. After listening to each tone through headphones, participants were asked to write down the pitch name (C, F#, E, etc.) on a response sheet. One point was given for each correct answer, and 0.75 point was given for each semitone error, but zero point was given for each error of more than a semitone. Finally, 20 students who scored above 32 (a score of 80% of the maximum) on each of two AP tests were designated AP possessors (Hutka & Alain, 2015; Kim & Knösche, 2017; Miyazaki, 2004b; Ross et al., 2004), while 20 students who scored below 20 (a score of 50% of the maximum) on the tests were considered non-AP possessors (Miyazaki & Rakowski, 2002; Miyazaki et al., 2018).

AP and non-AP possessors were matched for age, sex, and years of music training, but the evidential strength for *H*_0_ seemed to be different (see Table 1): There was moderate evidence for the absence of the effects of age and music training, but only weak evidence for the absence of the effect of sex. There was extreme evidence for *H*_1_ that AP possessors outperformed non-AP possessors on the two AP tests. The equivalence test was significant for sex (*z* = 3.44, *p* < .001), which means that we can reject the observed effect sizes as large or larger than *d* = 0.5. Yet, it was not significant for age, *t*(37.19) = 1.27, *p* = .107, or music training years, *t*(37.01) = 1.13, *p* = .134, which means that we cannot reject effect sizes as large or larger than *d* = 0.5 and need more data to prove the equivalence of the two groups. Ethical approval was granted by Shanghai Normal University in China, and all participants signed written informed consent and were paid for their participation.

**Table 1.** Participant Characteristics.

Variable	AP	Non-AP	*t*	*df*	*p*	*g*/Log *OR* (95% CI)	*BF*_10_
Age (years)	21.20 ± 1.61	21.35 ± 1.39	−0.32	37.19	.754	−0.10 [−0.72, 0.52]	0.32
Sex (male/female)	4/16	1/19	–	–	.342	−1.52 [–5.50, 0.92]	0.63
Years of music training	16.35 ± 1.87	16.10 ± 1.59	0.46	37.01	.651	0.14 [–0.48, 0.76]	0.34
AP Test 1 for pure tones (%)	88.66 ± 5.12	23.59 ± 9.07	27.95	30.00	<.001	8.66 [6.39, 10.92]	2.36 × 10^23^
AP Test 2 for piano tones (%)	97.56 ± 2.81	24.63 ± 7.34	41.50	24.45	<.001	12.86 [9.22, 16.39]	3.23 × 10^29^

*Note*. Values are *M* ± *SD*. AP = absolute pitch; *BF* = Bayes factor.

### Materials

**Figure 1.**
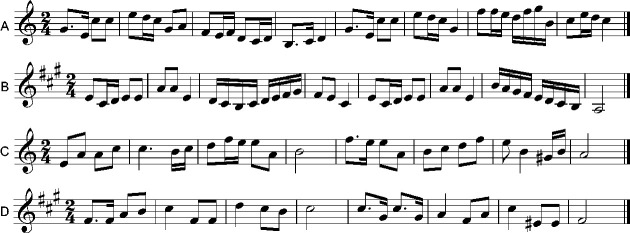
Examples of Melodies. Panel A: C major. Panel B: A major. Panel C: a minor. Panel D: f# minor.

The stimuli consisted of 24 original melodies with a duration of 10–20 s. These melodies were selected from Western classical music or European folk music along the structural regularities of tonal musical system. Half of the melodies were written in a major mode (six being in C major, and six being in D major, A major, E major, B♭ major, E♭ major, and A♭ major, respectively), and the other half were written in a minor mode (six being in a minor, and six being in b minor, f# minor, c# minor, g minor, c minor, and f minor, respectively; see Figure 1). To obtain a wider range of tension responses (Lerdahl & Krumhansl, 2007; Wong et al., 2009), each melody had five different endings with a tonic, two nontonic in-key notes, and two out-of-key notes, thus leading to 120 melodies. They were presented in the single-key condition (i.e., C major and a minor) or in the mixed-key condition (i.e., D major, A major, E major, B♭ major, E♭ major, A♭ major, b minor, f# minor, c# minor, g minor, c minor, and f minor). All melodies were generated with Overture 4.1 (GenieSoft, Inc., Summerville, SC, USA) and played with a grand piano sound using Pianissimo 1.0 (Acoustica, Inc., Oakhurst, CA, USA). Sound files were recorded with a sample rate of 44100 Hz, 16-bit resolution, and 705-kbps bit rate.

To derive participants’ ratings on musical tension from music characteristics, we computed the proportions of unstable melodic intervals for each melody with jSymbolic 2.2 (McKay et al., 2018). According to Costa et al. (2004), a sense of instability is related to a greater occurrence of minor seconds, tritones (also known as the augmented fourths), and compound intervals larger than the octave. Owing to a lack of larger intervals in the melodies, we thus computed the proportional occurrence of the first two intervals. In addition, we also extracted some psychoacoustic parameters for each melody with the MIR toolbox 1.7.2 (Lartillot et al., 2008). Based on prior research (Farbood & Price, 2017; Jiang et al., 2017; Parncutt et al., 2019), roughness, spectral flux, roll-off, and key clarity were included. Roughness is related to sensory dissonance (Parncutt & Hair, 2011); spectral flux refers to a measure of the fluctuation of the spectrum over time, with a low value causing relaxed feelings and a high value causing happy feelings (Laurier, 2011); roll-off is the frequency that splits the signal energy into two parts using a threshold in energy, with a low value for sad music (Laurier, 2011); key clarity estimates the strength of the best fitting key over time, with higher value indicating that music sounds steady and smooth (Yang & Chen, 2011). These musical features are shown in Table 2.

**Table 2.** Description of the Music Stimuli.

Variable	Single-key condition		Mixed-key condition	
	Major mode	Minor mode	Major mode	Minor mode
Interval category				
Minor second	12.90 ± 5.72	24.66 ± 6.49	13.33 ± 5.18	26.38 ± 8.08
Tritone	1.33 ± 2.85	1.29 ± 1.77	0.50 ± 1.14	1.23 ± 1.54
Acoustic parameters				
Roughness	4963.63 ± 3124.71	3758.56 ± 1301.86	5380.97 ± 1135.67	5396.22 ± 2508.21
Spectral flux	13.15 ± 3.25	11.48 ± 1.48	12.52 ± 1.32	12.05 ± 2.83
Roll-off	1436.84 ± 38.37	1398.09 ± 37.79	1593.97 ± 241.39	1477.62 ± 89.17
Key clarity	0.69 ± 0.07	0.66 ± 0.08	0.72 ± 0.11	0.69 ± 0.12

*Note*. Values are *M* ± *SD*.

### Procedure

Stimuli presentation was controlled by the software E-Prime 1.1 (Psychology Software Tools, Inc., Sharpsburg, PA, USA). The melodies were divided into two blocks: one block for melodies in the single-key condition and one block for melodies in the mixed-key condition. Within each block, melodies were played by Philips SHE1360 headphones in a pseudorandom order such that the same melodies with different endings were separated by at least three different melodies. Block order was balanced across participants.

In the tension perception task, after listening to each melody, participants were asked to rate the perceived tension of each melody as a whole by moving the needle of the continuous response digital interface dial (Madsen & Fredrickson, 1993), which ranged from “0” on the left side (least tension) to “255” on the right side (most tension). In the tension experience task, participants were required to rate the felt tension induced by the whole melody. To reduce the effect of familiarity, the experience task was conducted 6 months after participants completed the perception task. Prior to the experiment, participants were given two practice trials for each task and adjusted the sound volume to their most comfortable listening level.

### Data Analysis

We ran traditional null hypothesis significance testing using JASP (Version 0.13.1), where Welch’s *t* tests and Fisher’s exact test were performed on the demographic data, and multiple two-way analysis of variance (ANOVA) with key (single-key, mixed-key), and mode (major, minor) as the between-items variables were performed on the measures for music stimuli. In addition, a four-way ANOVA was performed on the ratings of musical tension, with group (AP, non-AP) as the between-subjects factor, and task (perception, experience), key (single-key, mixed-key), and mode (major, minor) as the within-subjects factors.

When the null hypothesis test is not significant, it is impossible to conclude the absence of an effect (Amrhein et al., 2019; Greenland et al., 2016; Keysers et al., 2020), because the design may be underpowered to detect the true effect (Harms & Lakens, 2018; Lakens, 2017). In other words, a nonsignificant result fails to diﬀerentiate between evidence of absence and absence of evidence (Dienes, 2014; van den Bergh et al., 2020). In this case, Bayes factors (*BF*s) and equivalence tests provide support for the lack of a meaningful effect (Anderson & Maxwell, 2016; Harms & Lakens, 2018; Lakens et al., 2020; Maxwell et al., 2015).

We ran Bayesian hypothesis testing and calculated *BF*s using JASP. The *BF* is the ratio of the probability of one hypothesis or model over another and can quantify the relative strength of evidence for two rival hypotheses (Brydges & Bielak, 2020; Love et al., 2019; Wagenmakers et al., 2018). *BF*_10_ denotes the odds ratio favoring *H*_1_ over *H*_0_, whereas *BF*_01_ denotes the odds ratio favoring *H*_0_ over *H*_1_. Typically, a *BF*_10_ of < 0.01 indicates *extreme*, 0.01–0.03 *very strong*, 0.03–0.10 *strong*, 0.10–0.33 *moderate*, and 0.33–1 *anecdotal* (i.e., weak, inconclusive) evidence for *H*_0_, and of 1 *no evidence* for *H*_0_ or *H*_1_, and of 1–3 *anecdotal*, 3–10 *moderate*, 10–30 *strong*, 30–100 *very strong*, and > 100 *extreme* evidence for *H*_1_ (Jeffreys, 1961; Lee & Wagenmakers, 2014). For example, *BF*_10_ =15 means that the data are 15 times more likely under *H*_1_ than under *H*_0_ and provide strong support for *H*_1_. To compute the *BF*, we first specified the competing hypotheses for the population standardized effect size δ: *H*_0_ postulates that there is no effect between groups or conditions (δ = 0), whereas *H*_1_ assumes that there is a true effect (δ  ≠  0). For Bayesian *t* tests, a Cauchy distribution centered on zero with scale *r* = 0.707 was used as a default choice for the prior distribution of *H*_1_, that is, *H*_1_: δ ∼ Cauchy(0, 0.707). For Bayesian contingency table, an independent multinomial sampling scheme (the row margins are fixed) with the default prior concentration of *a* = 1 was used such that *H*_1_ involves that there is a difference in sex ratio between groups. For Bayesian two-way ANOVAs, the default priors (fixed effects *r* = 0.5, random effects *r* = 1) were used to compute an inclusion *BF* (*BF*_incl_), which is the average posterior inclusion probability (i.e., *P*(*M*|data)) for all models that include the factor divided by that of their matched models that do not (Field et al., 2020; Keysers et al., 2020). The *BF*_incl_ can provide evidence for or against including an effect or interaction and be interpreted by the same thresholds for the strength of evidence as the *BF*_10_. For Bayesian four-way ANOVA, the default parameter priors (fixed effects *r* = 0.5, random effects *r* = 1, covariates *r* = 0.354) were used to compute the *BF*_incl_, while the subject factor was treated as a random effect. Regarding the ANOVAs, *H*_1_ refers to the inclusion of a main effect or an interaction. When there was no group difference, the same Bayesian *t* test was used to conduct a power analysis.

We also ran equivalence tests based on the two one-sided tests procedure (Lakens, 2017; Lakens et al., 2018; Meyners, 2012) using the TOSTER package (Version 0.3.4) in R (Version 4.0.2) and RStudio (Version 1.3.1073). To perform the equivalence tests, we first specified equivalence bounds based on the smallest effect size of interest (SESOI) that was deemed equivalent to the null value. Because of no theoretical predictions, practical importance, or related work in the literature, the SESOI had to be based on the benchmarks suggested by Cohen (1988). For differences between two independent groups or related conditions, we set the SESOI to a standardized effect size (*d* = 0.5). The value would then be used to determine a lower (Δ*_L_* = –0.5) and an upper (Δ*_U_* = 0.5) equivalence bounds. Next, two one-sided significance tests based on Welch’s *t* tests were conducted to examine whether the observed mean difference between groups or conditions (Δ) was larger than –0.5 or smaller than 0.5. In the two one-sided tests procedure, the null and alternative hypotheses were *H*_0_: Δ < –0.5 or Δ > 0.5, *H*_1_: –0.5 ≤Δ ≤0.5, respectively. If *p* values are below .05 for the two tests, then one can conclude that the observed effect is statistically equivalent to zero; but for simplicity, we reported only the one-sided test with the larger *p* value (Harms & Lakens, 2018; Lakens et al., 2020).

## Results

### Musical Features

#### Interval Category

As can be seen in Table 3, the two-way ANOVA on the occurrence of the minor second provided extreme evidence for an effect of mode, with there being ∼12% more minor seconds in minor melodies than in major melodies, but moderate evidence for the absence of an effect of key and an interaction between mode and key. The equivalence test for key yielded a significant result, *t*(116.75) = 2.08, *p* = .020, suggesting that the difference in percentages of the minor second between single- and mixed-key conditions was statistically equivalent to zero. A same ANOVA on the occurrence of the tritone provided moderate evidence for the absence of an effect of mode but weak evidence for the absence of an effect of key and an interaction effect. The equivalence test for mode yielded a significant result, *t*(109.60) = 1.74, *p* = .042, indicating that this effect was statistically equivalent to zero. But the equivalence test for key yielded a nonsignificant result, *t*(95.83) = −1.47, *p* = .072, indicating that this effect was not equivalent to zero.

**Table 3.** ANOVA Results and *BF*_incl_ Values for Different Effects in Music Features.

Variable	Effect	*F*(1, 116)	*p*	ω^2^	*BF*_incl_
Interval category					
Minor second	Key	0.83	.365	<.01	0.28
	Mode	110.63	<.001	.48	3.97 × 10^15^
	Key × Mode	0.30	.585	<.01	0.30
Tritone	Key	1.60	.208	<.01	0.40
	Mode	1.00	.320	<.01	0.30
	Key × Mode	1.19	.278	<.01	0.41
Acoustic parameters					
Roughness	Key	6.65	.011	.04	3.65
	Mode	2.23	.138	.01	0.55
	Key × Mode	2.35	.128	.01	0.66
Spectral flux	Key	<0.01	.962	<.01	0.19
	Mode	6.11	.015	.04	2.94
	Key × Mode	1.92	.169	.01	0.61
Roll-off	Key	24.31	<.001	.15	4795.90
	Mode	10.44	.002	.06	17.94
	Key × Mode	2.61	.109	.01	0.77
Key clarity	Key	3.58	.061	.02	0.96
	Mode	3.16	.078	.02	0.79
	Key × Mode	<0.01	.975	<.01	0.27

*Note*. *BF* = Bayes factor.

#### Acoustic Parameters

For roughness, the data provided moderate evidence for the main effect of key but weak evidence against the main effect of mode and the interaction between key and mode. The equivalence test for mode indicated that the observed effect was not statistically equivalent to zero, *t*(117.13) = −1.29, *p* = .100. For spectral flux, the data provided weak evidence for the effect of mode but moderate and weak evidence against the effect of key and the interaction between key and mode, respectively. The equivalence test for key indicated that this effect was statistically equivalent to zero, *t*(114.32) = −2.69, *p* = .004. For roll-off, the data provided substantial evidence for the effects of key and mode but anecdotal evidence against the interaction. For key clarity, the data provided moderate evidence against the interaction of key and mode but weak evidence against the effects of key and mode. Equivalence tests for the effects of key and mode were nonsignificant, respectively, *t*(102.12) = 0.86, *p* = .197 and *t*(117.06) = −0.97, *p* = .166.

Taken together, these results suggest that the minor melodies have more minor seconds than the major melodies. Meanwhile, the values of spectral flux, roll-off, and key clarity are lower in the minor melodies than in the major ones.

### Tension Ratings

**Figure 2.**
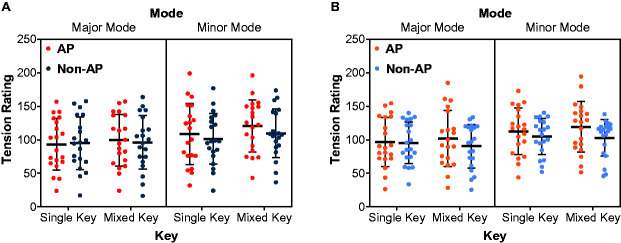
Mean Ratings of Tension as a Function of Key and Group for the Major and Minor Melodies. Panel A: The perceived tension. Panel B: The felt tension. Each dot represents data from an individual; error bars denote *M* ± *SD*. AP = absolute pitch.

**Figure 3.**
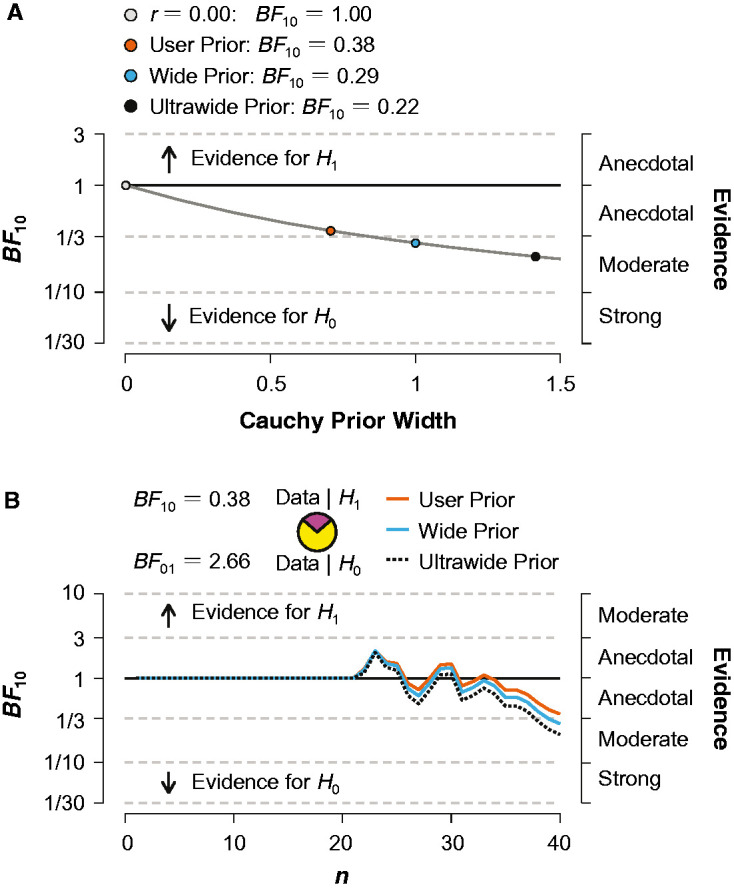
Results of Bayesian Analysis. Panel A: The *BF* as a function of the scale *r* of the Cauchy distribution, with a user prior (*r* = 0.707), a wide prior (*r* = 1.00), and an ultrawide prior (*r* = 1.41). Panel B: Development of *BF*s with increasing sample size. *BF* = Bayes factor.

Figure 2 shows the mean ratings of the perceived and felt musical tension by AP and non-AP groups in different conditions. The four-way ANOVA revealed extremely strong evidence for the presence of the effect of mode, *F*(1, 38) = 38.26, *p* < .001, ω^2^ = .04, *BF*_incl_ = 7.24 × 10^6^, with the major melodies receiving lower tension ratings than the minor ones. There was moderate evidence for the absence of the effect of task, *F*(1, 38) < 0.01, *p* = .990, ω^2^ < .01, *BF*_incl_ = 0.12, and weak evidence for the absence of the effects of key, *F*(1, 38) = 4.46, *p* = .041, ω^2^ < .01, *BF*_incl_ = 0.58, and group, *F*(1, 38) = 0.50, *p* = .486, ω^2^ < .01, *BF*_incl_ = 0.51. A Bayesian independent samples *t* test still showed inconclusive evidence for the lack of a group difference, *t*(37.37) = 0.70, *p* = .486, *BF*_10_ = 0.38, with median posterior δ = 0.17, 95% CI = [−0.37, 0.74]. To assess the robustness of the result, we repeated this analysis for different prior specifications as shown in Figure 3A. It is evident that, as the scale increases, the evidence in favor of the null grows. A sequential analysis was also performed to assess how the *BF* changes as sample size. This analysis demonstrates the sequential development of the evidence as the data accumulate and suggests that we need at least 40 participants per group to provide moderate evidence in favor of no group difference (see Figure 3B).

There was also moderate evidence for the absence of the interactions of Task × Mode, *F*(1, 38) = 0.01, *p* = .914, ω^2^ < .01, *BF*_incl_ = 0.18; Key × Mode, *F*(1, 38) = 3.42, *p* = .072, ω^2^ < .01, *BF*_incl_ = 0.27; Task × Group, *F*(1, 38) = 0.24, *p* = .626, ω^2^ < .01, *BF*_incl_ = 0.26; Task × Key × Mode, *F*(1, 38) = 2.41, *p* = .129, ω^2^ < .01, *BF*_incl_ = 0.24; Task × Key × Group, *F*(1, 38) = 0.37, *p* = .549, ω^2^ < .01, *BF*_incl_ = 0.31; Task × Mode × Group, *F*(1, 38) = 0.21, *p* = .651, ω^2^ < .01, *BF*_incl_ = 0.25; and Key × Mode × Group, *F*(1, 38) = 0.06, *p* = .810, ω^2^ < .01, *BF*_incl_ = 0.27, but weak evidence for the absence of the interactions of Task × Key, *F*(1, 38) = 1.95, *p* = .171, ω^2^ < .01, *BF*_incl_ = 0.38; Key × Group, *F*(1, 38) = 3.44, *p* = .072, ω^2^ < .01, *BF*_incl_ = 0.57; Mode× Group, *F*(1, 38) = 2.43, *p* = .127, ω^2^ < .01, *BF*_incl_ = 0.55; and Task × Key × Mode × Group, *F*(1, 38) = 0.02, *p* = .886, ω^2^ < .01, *BF*_incl_ = 0.35.

In addition, the equivalence test revealed that the effect of task was statistically equivalent to zero, *t*(39.00) = 3.15, *p* = .002, suggesting that we can reject the observed effect sizes more extreme than *d* = 0.5. Nevertheless, the effect of group was statistically not equivalent to zero, *t*(37.37) = −0.88, *p* = .193, suggesting that more data need to be collected.

In sum, these results consistently indicate that AP and non-AP possessors rate the major melodies less tense than the minor ones. Moreover, the two groups may be comparable during the perception and experience of musical tension.

## Discussion

To investigate whether AP is correlated with different musical tension processing, we tested AP and non-AP possessors on perceived and felt musical tension, an aspect of affective response to music. Although AP and non-AP possessors rated the major melodies as less tense than the minor ones, they seemed comparable on ratings of perceived and felt tension. These findings suggest that AP may be unrelated to the perception and experience of musical tension.

The first finding of our study is that AP and non-AP possessors rated the major melodies less tense than the minor ones. This result is in line with previous evidence that the minor triads or melodies express higher tension than the major triad (Lahdelma & Eerola, 2016) or melodies (Huynh et al., 2017) and that the minor music induces greater tension than the major music (Fang et al., 2017; Nielzen & Cesarec, 1982), which could be explained by two possibilities. According to the psychological model of tension and suspense, tension as an affective state usually originates from events associated with instability/dissonance/conflict or uncertainty that triggers predictive processes directed at future events of emotional signiﬁcance (Lehne & Koelsch, 2015). With regard to instability, the minor music excerpts have been found to be more unstable than the major ones (Lindström, 2006) and may result from a greater occurrence of minor seconds, tritones, and/or compound intervals larger than the octave (Bowling et al., 2010; Costa et al., 2004).^1^ Indeed, in our study, there were more minor seconds in minor melodies than in major ones. Moreover, the lower key clarity in the minor but not major music means higher tonal instability. Alternatively, the minor melodies relative to the major ones sound more sad and unpleasant (Bonetti & Costa, 2019; Fang et al., 2017; Lindström, 2006; Pittenger, 2002), which are relevant to a general feeling of uncertainty or insecurity (Parncutt, 2014). The uncertainty may be due to the fact that the minor triad and scale are generally more ambiguous and variable than the major ones (Parncutt, 2014) because, unlike the major scale, the minor scale has three competing forms: natural, harmonic, and melodic minor (Vuvan et al., 2011). Furthermore, it has been suggested that high spectrum flux is associated with feelings of happiness, while low roll-off is associated with feelings of sadness (Laurier, 2011). We also observed that major melodies are featured with high spectral flux and minor melodies with low roll-off. This further confirms that the major melodies in this study evoked a sense of happiness, while the minor melodies evoked a sense of sadness.

The second ﬁnding of our study is that AP may have no impact on the perception or experience of musical tension. Two previous studies have reported that AP possessors did not outperform non-AP possessors on tonality perception (Temperley & Marvin, 2008) and harmonic syntax processing (Coll et al., 2019). Our study extends these results to key aspects of high-level music processing and suggests a dissociation between AP and musical tension processing. However, it is contradictory to the other two studies having claimed that AP possessors exhibited superior performance in perceiving tonality (Marvin & Brinkman, 2000) and processing melodic and harmonic syntax (Jiang et al., 2010). The discrepant findings may be attributed to differences in task demands. Specifically, Marvin and Brinkman (2000) asked participants to name the key of a musical passage. Such task is required to determine the tonic and access the AP strategy. Thus, it is relatively easy for AP possessors than for non-AP possessors. By contrast, we made participants rate the levels of musical tension. Musical tension processing corresponds to the response to musical emotion, which is mostly governed by the sensitivity to changes of tonal pitch structure (e.g., harmonic progression) and does not require to use a certain pitch strategy. Therefore, AP and non-AP possessors can respond to musical tension using the knowledge of tonal pitch structure, which is acquired implicitly through everyday exposure to tonal music (Tillmann et al., 2000). Although AP possessors could build associations between pitch naming and emotional representations (Hsieh & Saberi, 2008), this emotional strategy might be not effective for tension processing, given that musical tension is related to the dynamic development of musical structures over time. In the study by Jiang et al. (2010), participants heard three melodic or chord sequences, with ending on a tonic/tonic chord or on a nontonic/nontonic chord, and determined whether the third sequence was identical in tonal stability to the first or second one. Because each sequence lasted within 20 s, and there was a silent interval of 3 s between two sequences, this task might result in a high working memory load. Considering that AP possessors have exhibited superior working memory for tonal and atonal melodies as compared with non-AP possessors (Hutka & Alain, 2015), it is not surprising that they perform better on such task. In contrast, participants in current study made tension judgments after the presence of each melody, which did not require a high working memory load. Hence, AP and non-AP possessors yielded similar ratings. Overall, the present findings suggest that the AP may not be associated with the processing of musical tension.

Another ﬁnding of our study is that there was no difference between perceived and felt tension ratings. This finding is incompatible with prior work showing that participants give higher arousal ratings in the emotion perception task than in the emotion experience task when music excerpts are chosen by the researchers (Kallinen & Ravaja, 2006; Salimpoor et al., 2009; Schubert, 2007). This discrepancy may arise from the differences in participants between the studies. Specifically, participants in those studies had little or no music training, while those in our study majored in music and mean reported 16 years of instrumental training. Indeed, research has demonstrated that people with more musical expertise reported more empathy than those with less one and were not able to distinguish between perceived and felt arousal (Egermann & McAdams, 2013).

In addition, perceived and felt tension ratings were lower for melodies in the single-key condition than melodies in the mixed-key condition, which is consistent with the finding of Jiang et al. (2016), who found that it was easier for participants to detect melodic structure violations in the single-key condition than in the mixed-key condition. This might be explained by the fact that the single-key condition results in the effect of an auditory sensory memory trace for in-key scale tones (Koelsch et al., 2007), whereas the mixed-key condition prevents accumulation effects on key structure (Jiang et al., 2016). Consequently, tonal expectancies to melodic development in the single-key condition should be stronger than those in the mixed-key condition and, in turn, decreased perceived and felt tension levels.

There are several limitations in this work. First, the sample size of the study is relatively small so that it is difficult to make a strong conclusion about the null effect of group difference. Second, the potential order effects were not balanced across the two tasks. Although the perception and experience tasks in this study were separated by 6 months, one may suspect that the unbalanced order has diminished the real effect (Gravetter & Forzano, 2018). However, it seems unreasonable to assume that the result of this study is distorted by the order effects, because the task order did have very little or no impact on the results of arousal (Schubert, 2007) and valence (Konečni et al., 2008; Schubert, 2007) ratings. Even so, future research is warranted to determine whether the effect of the task order is negligible. Last, there is a problem dividing the melodies into two blocks. The melodies should have been presented in four blocks, that is, single major key (C major), mixed major keys (D major, A major, E major, B♭ major, E♭ major, and A♭ major), single minor key (a major), and mixed minor keys (b minor, f# minor, c# minor, g minor, c minor, and f minor). This flaw has probably led to a weak evidence for the effect of key, thus calls for future research.

To conclude, the current study demonstrated that major music was less tense than minor music. Nonetheless, AP possessors may not differ from non-AP possessors when perceiving and experiencing musical tension. These findings imply that AP may not be associated with the processing of musical tension. Future studies should use a sufficient sample size to explore the relationship between AP and other higher level music processing such as musical meaning.
